# Heat Coma Temperature and Supercooling Point in Oceanic Sea Skaters (Heteroptera, Gerridae)

**DOI:** 10.3390/insects9010015

**Published:** 2018-02-03

**Authors:** Tetsuo Harada

**Affiliations:** Laboratory of Environmental Physiology, Graduate School of Integrated Sciences and Arts, Kochi University, Kochi 780-8520, Japan; haratets@kochi-u.ac.jp

**Keywords:** heat coma temperature, supercooling point, cross tolerance, sea skaters, large dataset

## Abstract

Heat coma temperatures (HCTs) and super cooling points (SCPs) were examined for nearly 1000 oceanic sea skaters collected from in the Pacific and Indian Oceans representing four *Halobates* species; *H. germanus*, *H. micans*, *H. sericeus*, and *H.* sp. Analysis was conducted using the entire dataset because a negative correlation was seen between the HCTs and SCPs in all four species. A weak negative correlation was seen between HCTs and SCPs with a cross tolerance between warmer HCTs and colder SCPs. The weakness of the correlation may be due to the large size of the dataset and to the variability in ocean surface temperature. The negative correlation does however suggest that oceanic sea skaters may have some form of cross tolerance with a common physiological mechanism for their high and low temperature tolerances.

## 1. Introduction

There are three main types of cold hardiness in insects [1]: (1) cold tolerance in which liquid inside cells does not freeze (instead ices making in liquids outside cells); (2) cold avoidance in which freezing of liquids inside and outside cells is prevented with a lower super cooling point; and (3) chill injury in which damage accumulates with prolonged exposure to low temperatures around 0 °C. Oceanic sea skaters (Heteroptera, Gerridae) are a biologically unique group among insects because they inhabit a wide range of water types from fresh water (e.g., *Metrocoris histrio*) to brackish water and sea water (coastal and pelagic). Because of this, they are particularly well-suited for research on insect adaption to dynamic changes in the environment. Oceanic sea skaters have a relatively high cool coma temperature (CCT; temperatures at which the body stem is cool damaged and sticks to the water surface and the insect can no longer stride) of 10–15 °C or higher. Their CCT may be much higher than their super cooling point (SCP; lowest temperature reached just before an exothermic event caused by the release of latent heat) [2,3]. Oceanic sea skaters may therefore be prone to less hardiness to lower temperatures than other insects with the three described ways to be hardy to lower temperature described above. In comparison, *Aquarius paludum* water striders that live in fresh water in a warm temperate zone in Kochi, Japan (33° N) have a lower lethal point of around −3 °C and SCP of around −10 °C, with coma occurring at about 0 °C [4], suggesting that adults of this species are prone to chill injury. Meanwhile, SCPs and lower lethal points were similar in nine species of semi-aquatic bugs inhabiting a cool temperature zone in south Bohemia, Czech Republic (49° N) [5].

Many oceanic sea skaters reach a cool coma at 10–15 °C or even higher [6]. The difference between cool coma temperature and SCP was larger for oceanic sea skaters (20–35 °C) than for fresh water striders (5–15 °C) [4,6]. The reason the lower lethal point is so much higher in oceanic sea skaters may be because the water surface temperature in the tropical ocean is very stable and fluctuates across a limited range of 23 to 31 °C. In fresh water semi-aquatic bugs, the lower lethal temperature is around 0 °C and their SCPs are from −6 °C to −8 °C on average, which is near to the lethal temperature [4]. On the other hand, oceanic sea skaters have much lower SCPs than coma (or lethal) temperature [3]. 

What is the biological significance of oceanic sea skaters having such low SCPs? As they inhabit sea water with 36‰, this would increase the salinity of hemolymph and the higher osmotic pressure would induce lower SCPs. Meanwhile, *Halobates micans* that inhabit the tropical Indian Ocean (around 8–10° S, 80–90° E) have extremely high heat coma temperatures (HCTs; temperature when the body stem is heat damaged and sticks to the water surface and the insect can no longer stride) of 41 °C on average [3]. This may be due to their original location at the origin of Madden–Julian Oscillation where intra-seasonal fluctuation of surface sea temperatures is the highest in the world (Yoneyama, personal communication). Absolutely no correlation was seen between HCTs and SCPS in oceanic sea skaters in that area [3]. The HCTs may be flexible and strongly affected by the large seasonal temperature fluctuations. Such flexibility could weaken the correlation. Although several environmental conditions (such as salinity level and dynamic seasonal temperature changes in the ocean) can modify HCTs and SCPs, one may predict that a negative correlation between HCTs and SCPs exists as a weak phenomenon common to all oceanic sea skaters. 

In a recent review article, Kaunisto et al. [7] described how several harsh environmental (biotic and abiotic) stressors have led to the evolution of tolerances to these multiple stressors in insects. One harsh environmental stressor for oceanic sea skaters may be intense solar radiation without cover to hide. One possible hypothesis might be that the evolution of tolerance to sun radiation through natural selection in oceanic sea skaters has led to the evolution of several other tolerances to higher and lower temperatures including lower SCP and starvation, for example. These cross tolerances could have co-evolved with a common underlying physiological mechanism.

The aim of this study is to determine whether the negative correlation between HCTs and SCPs is a general phenomenon. Analysis was performed on the HCTs and SCPs of around 1000 oceanic sea skaters obtained during 11 cruises. 

## 2. Materials and Methods

### 2.1. Samplings

Samplings were performed during 11 cruises from 2006 to 2013 on the research vessels TANSEIMARU (606t) (TH-09-20 cruise), HAKUHOMARU (3991t) (KH-06-02, KH-07-04, KH-10-04 and KH-12-02 cruises), and MIRAI (8687t) (MR06-05, MR08-02, MR09-04, MR10-03, MR11-07, and MR13-03 cruises), all owned by JAMSTEC (Japan Agency for Marine-Earth Science and Technology) (Table A1). Most sampling areas were in the tropical region of the Pacific (Table A1(A,B,D,E,H): 17° N to 0° N, 130° E to 156° E) and Indian (Table A1(C,I): 7° S to 8° S, 76° E to 83° E) Oceans. The sampling area was tropical and/or subtropical for two cruises (Table A1(J,K): 12 to 27° N, 135° E to 178° E) and temperate for two cruises (Table A1(F,G)). Three species, *H. germanus*, *H. micans*, and *H. sericeus*, were dominant in the tropical Pacific Ocean. Two, *H. germanus* and *H. micans*, were dominant in the tropical Indian Ocean (Table A1). 

An ORI (Ocean Research Institute, The University of Tokyo) net (3 m diameter, 6 m length) was used for sampling during the MR06-05 cruise. A small neuston net (75 cm × 55 cm × 100 cm square box type owned by the Ocean Research Institute) was used during the KH-07-04 cruise. Another size of neuston net (1.3 m diameter, 6 m length) was used for sampling during all other cruises. The ORI and neuston nets were trailed for 15 min, 3 to 18 times continuously at each station (in total three to 18 trials in one session) on the sea surface each night in the open water of the tropical Indian Ocean (Table A1(C)) and Pacific Ocean (Table A1(A,B,D,E,F,G,H,J,K)). Each trial comprised trailing for 15 min exclusively at night at a ship speed of 2.0 knots. All contents including sea skaters were trapped in a grey plastic bottle (20 cm length, 12 cm diameter cylinder) at the end of the nets. 

As the trailing of the nets may cause a high level of stress in the insects, all specimens were placed on dry white paper after collection to recover from physical paralysis due to the shock of the net trailing. About 60–80% of sea skaters recovered from paralysis within 5–20 min. When sea skaters were trapped in the jelly of a jelly fish, the jelly was removed from the body of the sea skater very carefully and quickly by hand for recovery from paralysis. Only specimens that recovered completely and resumed active striding on the sea water surface with a normal posture were used for HCTs and SCPs measurement. Specimens that recovered from paralysis were chosen randomly for the experiments. After recovery, specimens were transferred to a round-shaped transparent plastic aquarium (30 cm diameter, 15 cm height) for heat coma experiments (HCEs). 

### 2.2. Treatment of Specimens after Sampling

Adults and first to fifth instars recovered from paralysis due to the trailing. These sea skaters were kept on sea water in numerous semi-transparent (to shut out the view from inside) white cube aquaria (30 cm × 30 cm × 40 cm) in the ship laboratory. These specimens were used for measurements of HCTs and SCPs. Each aquarium contained 10–20 adults and/or larvae of *Halobates*. Both the room temperature and sea water temperature in the aquaria were kept at 26 °C to 29 °C, similar to the surface temperature at the sampling sites. The experimental adult and larval specimens were kept in the aquaria at least 9 h after collection before the HCEs to allow for adaptation to the laboratory environment. Some *Halobates* specimens were not used in those experiments the day after collection and instead were kept in the aquaria with foods as adult *Lucillia illustris* for more than 12 h after collection. Although sea skaters would likely feed on zooplankton in their natural habitat [8], they were fed the adult flies. These specimens were supplied for the measurement of HCTs two days or later after collection. For each insect with 12 h starvation, heat coma experiments (HCEs) were conducted and then followed up by measurement of SCPs. Specimens were starved for 12 h because the substances derived from food in the alimentary canal could potentially cause an ice nucleus if the HCTs and SCP measurements were performed right after feeding. SCPs were measured just after the HCEs in order to examine the relationship between HCTs and SCPs.

### 2.3. Measurement of HCTs and SCPs

In a semi-transparent rearing aquarium (30 cm diameter, 15 cm height), sea skaters were reared on sea water at the same temperature (26 °C to 29 °C) in the ship laboratory before the HCEs and subsequent SCP measurements. Four or five layers of cardboard were wrapped around the round-shaped transparent experimental aquarium to function as an insulator. Around 10 individuals in the adult or larval stage were moved from the rearing aquarium to the experimental aquarium. The temperature was increased stepwise by 1 °C every hour until heat comas (HCs) occurred in all experimental specimens. The temperature was controlled very precisely with a handheld on–off switch to keep it within ±0.3 °C of the current water temperature. Hand stirring with a 10 cm long, 5 mm diameter air tube with a 3 cm diameter ball stone at the end was performed to effectively keep precise control of the temperature. Semi heat coma temperatures (SHCTs) and HCTs were defined as follows and recorded. When semi heat coma (SHC) occurs, little or no movement by the specimen is observed on the water surface for more than three seconds. When heat coma (HC) occurs, the ventral surface of the body of the specimen sticks to the sea water film and the specimen can no longer stride.

The body temperature, including hemolymph, should be similar to the surface temperature of the water as the body was positioned only one mm above the water surface. After heat coma at an extremely high temperature above 42 °C, all specimens died within two hours from severe damage [9]. The procedure for measuring thermoregulation on the vessels was somewhat difficult as few devices for measuring temperatures of various body parts could be brought on board.

Measurement of SCPs was performed on specimens of the four species of oceanic sea skaters—*Halobates germanus*, *H. micans*, *H. sericeus*, and *H*. sp.—that were still in a coma immediately after the measurement of HCTs. The insects were still in a coma when SCP measurement started. The surface of each adult was dried with filter paper and a thermocouple made of nickel and bronze was attached to the abdominal surface of the thorax. The thermocouple was then connected to an automatic digital temperature recorder (Digital Thermometer, Model 10, Yokogawa Co., Ltd., Musashino, Japan). The thermocouple was attached to the ventral surface of abdomen and fixed securely in place with a type of adhesive tape. The specimen attached to the thermocouple was placed in a compressed Styrofoam box (5 cm × 5 cm × 3 cm). This cover was again covered with larger insulating compressed Styrofoam. The double cover by compressed Styrofoam kept the cooling rate to about 1 °C/min for recording the SCPs in a freezer set to −35 °C. The lowest temperature reached just before an exothermic event occurred due to release of latent heat was regarded as the SCP [10]. All tested specimens died from body freezing when the SCPs were measured.

### 2.4. Statistical Analysis

**Figure 1:**
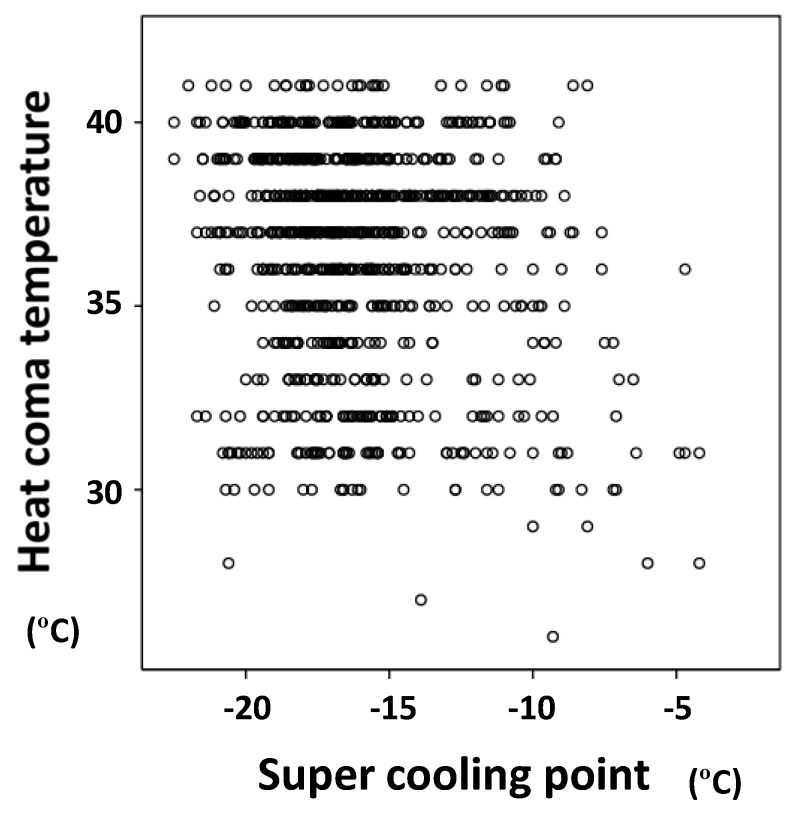
Negative correlation between heat coma temperatures (HCTs) and super cooling points (SCPs) in four species of oceanic sea skaters—*Halobates micans*, *H. germanus*, *H. sericeus*, and *H.* sp.—inhabiting temperate to tropical regions of the Indian and Pacific Oceans (Pearson’s correlative analysis: *r* = −0.178, *p* < 0.001, *n* = 1004).

The data was analyzed with SPSS 12.0 statistical software. Three-way analysis of variance (ANOVA) was performed on the relationships among species, sex, and stage and each measurement (SHCTs, HCTs, GTsHC, and SCPs) in around 1000 specimens of the four species of oceanic sea skaters, *Halobates germanus* (*H.g*.), *H. micans* (*H.m.*), *H. sericeus*, and *H.* sp. (Table 1 and Table 2). Pearson’s correlation analysis was performed between HCTs (SHCTs) and SCPs (Figure 1). Latitude of sampling place was excluded from the analysis factors because the relationships between HCTs and latitude and between SCPs and latitude were found to be too complex (fluctuation of SCPs in accordance with latitude) when one-way ANOVA was performed. 

## 3. Results

### 3.1. Samplings

In the tropical area far west of the Indonesian islands, only *H. micans* and *H. germanus* were collected during the KH-07-04 and MR11-02 cruises (Table A1(C,I)). Among *Halobates* individuals collected, four species—*H. germanus*, *H. micans*, *H. sericeus*, and *H.* sp—were used for the measurement of HCTs and SCPs (Table A1). The area of the western and central Pacific Ocean that ranged from 0° N to 10° N and 130° E to 160° E was inhabited by four oceanic sea skaters, *H. germanus, H. micans, H. sericeus*, and *H.* sp. (Table A1(A,D,E)). Those four species were also collected in October from the *Kuroshio* current (Table A1(F)). The *Kuroshio* current could have transferred the four species to a relatively higher latitude of 30° N to 40° N. Among the four species of oceanic sea skaters, *H. sericeus* inhabited the subtropical area that has a higher latitude of 20° N to 30° N in the relatively eastern area of 140° E to 170° W in the Pacific Ocean (Table A1(G,J)). The four species were collected in the subtropical area of 24° N in the Western Pacific area of 135° E (Table A1(K)). 

### 3.2. HCTs and SCPs

Based on a dataset of around thousand specimens, the averages of SHCTs, HCTs, GTsHC, and SCPs were around 33, 35, 7, and −16 °C, respectively (Table 1). The HCTs of third instar nymphs were significantly lower than those of adults (*T*-test, *t*-value = −2.001, N = 7 (third instars) and 1270 (adults), df = 1275, *p* = 0.046) (Table 1). The HCTs and SCPs of fifth instar larvae tended to be lower and higher, respectively, than those of adults (*T*-test, HCTs: *t*-value = −1.751, N = 148 (fifth instars) and 1270 (adults), df = 1416, *p* = 0.08, SCPs: *t*-value = −1.806, N = 110 (fifth instars) and 1034 (adults), df = 1142, *p* = 0.071) (Table 1).

There were no significant differences due to sex or stage (Table 2). However, there were significant interspecies differences in HCTs, SHCTs, GTsHC, and SCPs (Table 2). For example, the SCPs of *H. micans* were slightly lower by 0.5 °C than compared to the other three species. 

There was no significant correlation between SHCTs and SCPs (Pearson’s correlation test: *r* = −0.066, *p* = 0.091, n = 1004). However, significant negative correlations were seen between HCTs (GTsHC) and SCPs (HCT: *r* = −0.178, *p* < 0.001, n = 1004; GTHC: *r* = −0.157, *p* < 0.001, n = 1004) (Figure 1).

The mean HCTs of larvae were 33.0 °C to 35.3 °C, which was significantly lower than the mean HCTs of adults by 1 to 2 °C (35.7 °C), while the SCPs of larvae were similar to those of adults (Table 2). Three species—*H. germanus, H. micans* and *H. sericeus*—had similar mean HCTs of 35.4 °C to 35.9 °C that were significantly higher than the mean HCTs of the undescribed species, *H.* sp., that was 33.4 °C (Table 3). In contrast, the mean SCPs of *H.* sp. was around −18.0, which was lower than the mean SCPs of the other three species (−15.8 °C to −16.6 °C) (Table 3). During the cruise of MR11-07, the effects of experiencing heat coma (HC) on SCPs were examined (Table 3). There were no effects of HC on SCP in larvae. However, adults who experienced HCs showed significantly higher SCPs than specimens who had not experienced HCEs before measuring SCPs (Table 3). 

**Figure 2:**
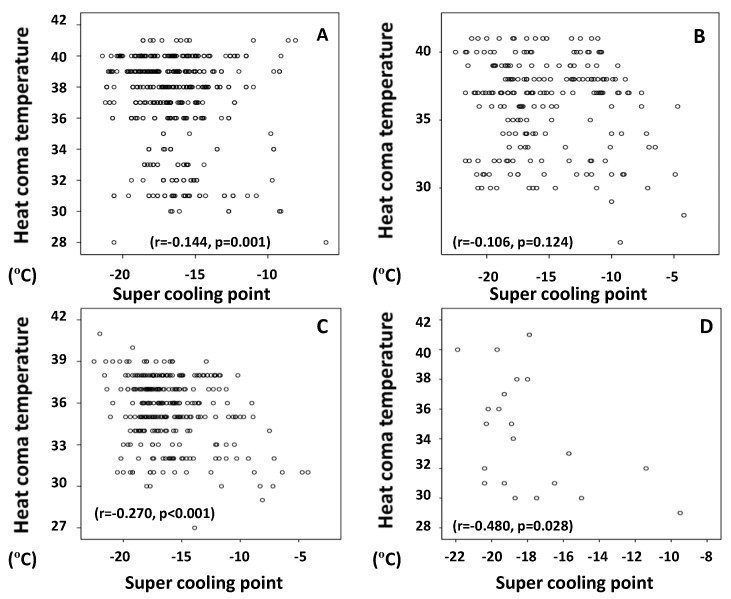
Negative correlation between heat coma temperatures (HCTs) and super cooling points (SCPs) in each of four species of oceanic sea skaters—*Halobates micans* (**A**), *H. germanus* (**B**), *H. sericeus* (**C**), and *H.* sp. (**D**)—inhabiting the temperate to tropical region of the Indian and Pacific Oceans.

The above correlations may only be significant due to the increased power of correlation of the large sample size (N > 1000) [11]. However, the finding remains that the negative correlation between HCTs and SCPs was stronger in *Halobates sericeus* and *H.* sp. than in *H. micans* and *H. germanus* (Figure 2).

## 4. Discussion

In the relatively large dataset of around one thousand specimens, there was a significant negative correlation between heat coma temperatures (HCTs) and super cooling points (SCPs) that may be a marker of cold tolerance. However, the correlation was very weak overall. Analysis of each of the four species showed a relatively high correlation between HCTs and SCPs in *Halobates sericeus* and *H.* sp. This correlation suggests that there may be a common mechanism underlying heat and cold tolerance. As a similar phenomenon, Salin [12] showed another example of cross tolerance based on an experiment. 

This experiment showed higher super cooling points and lower high-temperature stupor points under 0% relative humidity (RH), but lower super cooling points and high-temperature stupor points under 100% RH in the adult lesser mealworm, *Alphitobius diaperinus* (Coleoptera, Tenebrionidae) [12]. 

Boardman et al. [13] recently proposed physiological and molecular mechanisms associated with cross tolerance between hypoxia and temperature stresses in *Thaumatotibia leucotrea* (Lepidoptera, Tortricidsae). A common change after exposure to low and high temperatures was an increase in fatty acid chain length. A similar molecular mechanism could explain the negative correlation in HCTs and SCPs observed in the present study. The negative correlation could result from a molecule base mechanism that includes a cross tolerance related substance, especially in *Halobates sericeus* that inhabits a higher latitude range and would be more likely to develop a common mechanism for heat and cool tolerance. 

Despite very low P-values in regression A and C in Figure 2, there is a weak correlative relationship between HCTs and SCPs. The strongest correlation shown in Figure 2 was for D with *H.* sp. at *r* = −0.480, and even that may be considered weak. That said, this finding should not be discarded and may be a starting point. Table 3 shows one weakness of the dataset; SCPs suffered for adult *H. micans* in that they were warmer than they should be (imparting less cold protection) when that particular test insect was put in an HCE prior to measuring SCPs. This finding supports one of my main hypotheses that the physiological mechanisms regulating HCTs and SCPs are probably related and/or dependent on each other.

One possible area of future research would be to determine the correlations between HCTs and SCPs in separate groups of insects from different populations/species. If each insect needs to have two data measurements for use in the correlation analysis, then this cannot be avoided. An alternative may be to summarize the HCTs and SCPs for separate groups of insects from many different populations or species and then perform the correlation on those populations. This has not yet been carried out. The data may be substantial and I have shown a weak correlation between the two values. This weakness may be a topic for future research.

Regarding the radiation temperature that affects the body surface positioned on the ocean surface, tropical populations of oceanic sea skater species—*Halobates germanus*, *H. micans*, and *H. sericeus*—inhabiting the tropical Indian and Pacific Oceans may be exposed to temperatures as harsh as 40 °C or higher with no hiding places. The finding from the present study that many individuals had high heat coma temperatures of more than 40 °C is logical considering their ability to survive harsh radiation of sunlight in the tropical area. 

The above results may agree with the hypothesis of the present study that evolution of tolerance to sun radiation through natural selection in oceanic sea skaters may have led to the evolution of a lower SCP as a part of the mechanism for lower temperature tolerance, because the physiological mechanism of heat tolerance could be shared with the mechanism for SCP.

## 5. Conclusions

Significant negative correlations were seen between heat coma temperatures (HCTs) (or gap temperatures for heat coma: GTsHC) and super cooling points (SCPs) in oceanic sea skaters based on data from around 1000 specimens. However, the correlation is weak, possibly due to the large sample size or the wide variability of ocean surface temperatures. The negative (albeit weak) correlation observed in this study suggests that oceanic sea skaters may possess some form of cross tolerance that may be due to a common physiological mechanism between heat and cold tolerances. 

## Figures and Tables

**Table 1:** Comparisons of heat coma temperatures (HCTs) and super cooling points (SCPs) between five instars of larvae and adults and between species of individuals of *Halobates* in the Pacific and Indian Oceans.

	**First Instar**	**Second Instar**	**Third Instar**	**Fourth Instar**	**Fifth Instar**	**Adults**
HCT	33.00 (1)	34.00 ± 2.83 (2)	33.42 ± 2.07 (7)	34.00 ± 1.89 (15)	35.32 ± 3.42 (148)	35.79 ± 3.12 (1270)
SCP	-	-	−18.65 ± 4.31 (2)	−19.4 (1)	−16.81 ± 2.32 (110)	−16.28 ± 3.06 (1034)
	***Halobates germanus***	***H. micans***	***H. sericeus***	***H.* sp.**		
HCP	35.6 ± 3.52 (253)	35.93 ± 3.22 (830)	35.43 ± 2.44 (330)	33.64 ± 3.86 (33)		
SCP	−15.79 ± 3.94 (244)	−16.64 ± 2.42 (580)	−16.06 ± 3.01 (303)	−17.98 ± 3.00 (21)		
**Two-Way ANOVA (Relationship between Stage (and species) and HCT (and SCP)**						
	**Df**		**F-Value**		**P-Value**	
	**HCT**	**SCP**	**HCT**	**SCP**	**HCT**	**SCP**
Stage	5	3	3.44	1.432	0.004 **	0.232
Species	1	1	18.88	3.530	<0.001 ***	0.061

*: 0.05 > *p* > 0.01, **: 0.01 > *p* > 0.001, ***: 0.001 > *p*.

**Table 2:** Three-way ANOVA (species sex and stage) on the relationship between one of species, sex, and stage and semi heat coma temperatures (SHCTs), heat coma temperatures (HCTs), gap temperatures for heat coma (GTsHC) and super cooling points (SCPs) in the four species of oceanic sea skaters, *Halobates micans* (*H.m*.), *H. germanus* (*H.g.*), *H. sericeus* (*H.s.*), and *H.* sp. in a dataset of several hundred to a thousand specimens.

Three-Way ANOVA												
	df				F-Value				P-Value			
	HCTs	SHCTs	GsTHC	SCPs	HCTs	SHCTs	GsTHC	SCPs	HCTs	SHCTs	GTsHC	SCPs
Species	3	3	3	3	3.898	3.288	2.959	9.992	0.009 **	0.020 *	0.031 *	<0.001 ***
Sex	1	1	1	1	0.10	0.459	0.102	1.508	0.919	0.498	0.749	0.220
Stages	5	5	5	3	3.440	1.002	1.695	1.432	0.004 **	0.415	0.133	0.232

*: 0.01 < *p* < 0.05, **: 0.001 < *p* < 0.01, ***: *p* < 0.001; *: Degree of freedom of the denominator was 9.721 for HCTs, 12.754 for SHCTs, 8.430 for GTsHC and 9.075 for SCPs.

**Table 3:** Effect of heat coma temperature (HCT) measurement on the subsequent measurement of super cooling points (SCPs) and increased temperature at SCPs (ITSCPs) in *Halobates micans* during the cruise MR11-07 (Table A1(I))*.* Data on specimens collected at around 08°00′ S, 080°30′ E was analyzed. Experiments were performed during the period from 1 to 23 October 2011 in wet laboratory 2 of R/V Mirai (Mean ± SD(n)).

Effects of HC exp.	Stage			
	Fifth Instar		Adult	
	SCPs	ITSCPs	SCPs	ITSCPs
After HC exp.	−16.4±2.6 (27)	7.3±2.4 (27)	−16.1±2.7 (108)	6.7±2.3 (108)
Without HC exp.	−16.4±1.9 (13)	7.3±1.9 (13)	−18.4±2.0 (48)	7.3±2.0 (48)
Mann–Whitney U-test				
Z	−0.231	−0.448	−4.897	−1.74
P	0.817	0.669	<0.001 ***	0.082

*: 0.05 > *p* > 0.01, **: 0.01 > *p* > 0.001, ***: 0.001 > *p*.

## Appendix A

**Table A1:** The sea area, season, and year of specimens collected during the cruises to examine semi heat coma temperatures (SHCTs), heat coma temperatures (HCTs), gap temperatures for heat coma (GTsHC) and super cooling temperatures (SCPs) in the oceanic sea skaters, *Halobates*.

	Cruises	Latitude	Longitude	Date	Species *	Experiments
A.	MR06-05 cruise	8°00′N–	130°04′ E–	24 December–6 January	*H.m.*, *H.g.*,	SHCTs, HCTs,
		2°00′ N	138°00′ E	2006–2007	*H.s.*, *H.* sp.	GTsHC
B.	KH-06-02 cruise	17°00′ N–	142°00′ E–	18–27 August	*H.m.*, *H.g.*	HCTs
		12°00′ N	137°00′ E	2006	*H.s.*	
C.	KH-07-04 cruise	6°36′ N–	85°59′ E–	24–30 December	*H.m.*	SHCTs, HCTs,
		6°36′ S	76°37′ E	2007		GTsHC
D.	MR08-02 cruise	17°21′ N–	130°04′ E–	1–27 June	*H.m.*,* H.g.*,	SHCTs, HCTs,
		5°60′ N	135°00′ E	2008	*H.s.*, *H.* sp.	GTsHC
E.	MR09-04 cruise	10°00′ N–	156°00′ E–	12–19 November	*H.m.*, *H.g.*,	SHCTs, HCTs,
		0°00′ N	155°00′ E	2009	*H.s.*	GTsHC, SCPs
F.	TH-09-20 cruise	34°27′ N–	140°00′ E–	2–13 October	*H.m.*,* H.g.*	SHCTs, HCTs,
		30°10′ N	129°21′ E	2009	*H.s.*, *H.* sp.	GTsHC SCPs
G.	KH-10-04 cruise	34°43′ N–	140°14′ E–	2–7 September	*H.s.*	SHCTs, HCTs,
		19°33′ N	164°44′ W	2010		GTsHC SCPs
H.	MR10-03 cruise	7°50′ N–	139°31′ E–	9 May–20 June	*H.m.*	SHCTs, HCTs,
		5°01′ N	140°12′ E	2010		SCPs, GTsHC
I.	MR11-07 cruise	1°56′ S–	80°30′ E–	28 September–23 October	*H.m.*,* H.g.*	SHCTs, HCTs,
		8°00′ S	83°24′ E	2011		SCPs, GTsHC
J.	KH-12-02 cruise	27°12′ N–	169°24′ E–	26 February–1 March	*H.s.*	SHCTs, HCTs,
		24°30′ N	177°32′ W			GTsHCs, SCPs
K.	MR13-03 cruise	24°12′ N–	135°00′ E–	6–30 June	*H.m.*, *H.g.*	HCTs, GTsHC,
		12°12′ N	138°0′ E	2013	*H.s.*, *H.* sp.	SCPs

* Species used for HCTs and SCPs measurements: *H.m.*: *Halobates micans*; *H.g.*: *Halobates germanus*; *H.s.*: *Halobates sericeus*; *H.* sp.: *Halobates* sp.
